# Endometrial Microbiota and Immune Tolerance in Pregnancy

**DOI:** 10.3390/ijms24032995

**Published:** 2023-02-03

**Authors:** Annalisa Inversetti, Enrica Zambella, Alice Guarano, Marinella Dell’Avanzo, Nicoletta Di Simone

**Affiliations:** 1 Department of Biomedical Sciences, Humanitas University, Via Rita Levi Montalcini 4, Pieve Emanuele, 20072 Milan, Italy; 2 IRCCS Humanitas Research Hospital, Via Manzoni 56, Rozzano, 20089 Milan, Italy; 3 Humanitas San Pio X, Via Francesco Nava 31, 20159 Milan, Italy

**Keywords:** endometrium, microbiome, pregnancy, implantation

## Abstract

Recent studies have demonstrated that the uterus has its own microbiota. However, there is no consensus on endometrial microbiota composition, thus its role in the healthy uterine environment is still a frontier topic. Endometrial receptivity is key to embryo implantation, and in this specific context immunological tolerance against fetal antigens and the tightly regulated expression of inflammatory mediators are fundamental. According to recent evidence, endometrial microbiota may interact in a very dynamic way with the immune system during the peri-conceptional stage and later during pregnancy. For this reason, a condition of dysbiosis might lead to adverse pregnancy outcomes. The aim of this review is to summarize the evidence on the molecular mechanisms by which the endometrial microbiota may interact with the immune system. For this purpose, the link between dysbiosis and reproductive disorders, such as infertility, recurrent pregnancy loss (RPL), and preterm birth, will be discussed. In conclusion, the most recent findings from molecular analyses will be reported to illustrate and possibly overcome the intrinsic limitations of uterine microbiota detection (low endometrial biomass, high risk of contamination during sampling, and lack of standardization).

## 1. Introduction

The human microbiome is a collection of different microbes that inhabit the entire human body, and its role in physiology and disease is a topic of increasing interest in the scientific community. The Human Microbiome Project was one of the largest studies to describe all the microorganisms populating different sites (airways, oral cavity, skin, gastrointestinal tract, and urogenital tract) in 300 healthy volunteers from the United States (USA) with the aim of enrolling a sample population that was reasonably heterogenous in terms of ethnicity and other demographic features [1]. Historically, the gastrointestinal microbiota has been more fully investigated compared to the female reproductive tract microbiota, due to the less invasive sampling techniques and the common belief that the upper reproductive tract (URT) is a sterile environment. Nevertheless, the first proof of endometrial colonization in healthy women arrived in 1985, and it has been subsequently demonstrated that the female reproductive tract microbiota represents about 9% of the entire microbiota in women [2,3]. Thanks to the development of gene sequencing technologies, several studies of uterine microecology have been conducted, and the role of microbiota in reproductive health and endometrial disease has been investigated. According to recent evidence, the endometrium enables the growth of symbiotic bacteria which play a dual role:

1. Provide protection from tissue infection by competing with pathogenic bacteria;
2. Ensure immune-cell activation, strengthening the barrier of the endometrium, and promoting endometrial repair and angiogenesis [4,5].

Therefore, the dynamic balance of uterine microecology depends on the relationship between the endometrial microbiota, the immune system, and the endometrium, in both physiological and pathological states [6,7,8,9,10]. Moreover, these three factors all seem to be involved in embryo implantation, which remains one of the most challenging topics. As a matter of fact, the success of this process depends on both embryo quality and endometrial acceptability. Implantation failures occur due to low-quality embryos and poor endometrial receptivity [11,12]. It was demonstrated in many infertile women undergoing treatment with assisted reproductive technology (ART) that pregnancy failure recurs with repeated in vitro fertilization (IVF) cycles, even if the transferred embryos are of high quality [13]. Indeed, several local changes that lead to endometrial decidualization are required to enhance blastocyst implantation [14]. Several components, such as the cellular immune response and microbiome composition, can affect endometrial receptivity [15,16,17,18,19]. The aim of this review is to evaluate the possible role of the endometrial microbiome as an immune modulator in the phase of implantation and during pregnancy. Furthermore, the correlations between endometrial dysbiosis and adverse obstetric outcomes, such as infertility, recurrent pregnancy failure, and preterm labor, are investigated. 

## 2. Endometrial Microbiota and Its Variation during Lifespan

### 2.1. The Beginning—Initial Studies on the Topic: Emerging Differences Comparing Vaginal and Endometrial Microbiomes

Most of the initial studies detected *Lactobacillus* (L.) spp., *Gardnerella vaginalis, Enterobacter* spp., and *Mycoplasma hominis* through transcervical sampling [20,21]. Later, Mitchell et al. found a great abundance of *L. iners, Prevotella* spp., and *L. crispatus* in 95% of vaginal swabs obtained from 58 women undergoing hysterectomy for non-cancer indications [22]. However, less bacterial DNA in upper genital tract (URT; namely, the endocervix and endometrium) samples was obtained [22]. These findings were confirmed by Chen et al., who revealed that the vaginal sites contained about four orders of magnitude more bacteria than the endometrial sites [10]. Moreno and colleagues focused their interest on the similarity—but not perfect correspondence—between the intraindividual vaginal and the endometrial microbiome, highlighting that in approximately 20% of the subjects, the endometrial and vaginal microbiomes differed in terms of either the bacterial taxa identified or the relative abundances in which they were represented in both types of samples [9]. Further studies demonstrated that *Lactobacillus* was the most represented genus in endometrial samples [23], while other genera commonly detected were *Flavobacterium* [23], *Bifidobacterium, Gardnerella, Prevotella*, and *Streptococcus* [24]. 

### 2.2. Results from an Infertile Population and Women Undergoing ART Therapy

Sampling of a population undergoing ART therapy gave the unique opportunity to analyze the endometrial microbiome in an interesting temporal window (during embryo transfer) and anatomical site (from the embryo transfer catheter). 

Recent studies, reported in detail in Table 1, highlighted a potential association between a state of dysbiosis and the success of the ART procedure. Whether this association is supported by evidence is still a matter of debate, because the role of endometrial microbiome composition in subtypes of infertile patients has not yet been fully elucidated.

Firstly, a certain number of studies on this specific population revealed the possibility of *Lactobacillus* dominance [25,26,27,28,29]. 

Subsequent studies [30,31] did not confirm the *Lactobacillus* dominance that was first hypothesized. Higher diversity seemed to be associated with better outcomes after IVF procedures [31]. In conclusion, these contrasting findings highlighted several aspects: first, the need for a better understanding of endometrial microbiome composition and function in healthy women and its possible role in endometrial functions before studying its impact on the success of ART procedures; second, the relevance of subdividing the study population according to the cause of infertility; third, the need for standardized sampling methods.

**Table 1:** Endometrial microbiome composition in infertile women, including patients undergoing IVF or with a history of recurrent implantation failure (RIF).

Author, Year	Country	Number and Type of Patients	Sample (Method)	Findings
Moore, 2000 [32]	USA	91 women undergoing IVF	ET (Embryo transfer catheter tips)	Live birth rate (LBR): Significantly higher when associated with isolated H_2_O_2_-producing *Lactobacillus* vs. significantly lower with isolated *Streptococcus viridans*
Moreno, 2016 [24]	Spain	35 infertile women undergoing IVF	EF (Aspiration with catheter)	3 poor DNA quality 17 LDM 15 NLDM associated with: significantly lower implantation, pregnancy, ongoing pregnancy and LBR
Tao, 2017 [25]	USA	70 women undergoing IVF	ET (Embryo transfer catheter tips)	90% *Lactobacillus* abundance in 33 patients (47.1%)
Kyono, 2018 [26]	Japan	102 infertile women: 79 undergoing IVF 23 not undergoing IVF 7 controls	EF (IUI catheter)	90% *Lactobacillus* abundance in: 30 IVF patients (38%) 17 non-IVF patients (73.9%) 6 controls (85.7%)
Wee, 2018 [27]	Australia	16 infertile women; 15 controls	ET (Biopsy during hysteroscopy)	*Lactobacillus* more abundant in controls
Kyono, 2019 [30]	Japan	92 women undergoing IVF	EF (IUI catheter)	LDM in 47 cases (51.1%); NLDM in 45 cases (48.9%)
Kitaya, 2019 [33]	Japan	28 women with history of RIF (RIF group) vs. 18 infertile women undergoing IVF (non-RIF group)	EF (Curette)	Higher α-diversity in endometrium compared to vagina in both groups LDM: 18/28 (64.3%) RIF group 7/18 (38.9%) infertile group *Gardnerella:* 11/28 (39.3%) RIF group 5/18 (27.7%) control group *Burkholderia:* 7/28 (25%) RIF group 0/18 control group
Carosso, 2020 [28]	Italy	15 infertile women undergoing IVF pre- and post-COS	ET (Embryo transfer catheter tips)	*Lactobacillus* reduced post-COS *Prevotella* significantly increased post-COS *Atopobium* significantly increased post-COS
Kadogami 2020 [34]	Japan	392 women with history of RIF	EF (Pipette)	LD: 216/392 (55.1%) NLD: 176/392 (44.9%) with *Gardnerella* (the most abundant), *Atopobium*, *Streptococcus*, and *Prevotella*
Riganelli, 2020 [35]	Italy	34 infertile women undergoing ART	ET (Pipelle catheter covered by the IUI catheter)	Non-pregnant women (30/34) with increased levels of *Lactobacillus* species and significantly increased relative abundances of *Kocuria dechangensis*
Diaz-Martinez, 2021 [36]	Spain	48 infertile women undergoing IVF	ET (Brush)	*Delftia* spp., *Anaerobacillus* spp., and *Ralstonia* spp. more abundant in endometrium compared to vagina *Lactobacillus* spp., *Gardnerella* spp., *Burkholderia* spp., and *Anaerobacillus* spp. more abundant in pregnant women *Streptococcus* spp., *Ralstonia* spp., *Prevotella* spp., and *Delftia* spp. more abundant in non-pregnant women In RIF patients: Higher abundance of Prevotella, Lactobacillus helveticus, and Sneathia amnii Lower abundance of Ralstonia
Ichiyama, 2021 [37]	Japan	145 women with history of RIF 21 controls	ET (Pipette)	14 genera, including *Atopobium, Burkholderia, Delftia, Gardnerella,* and *Prevotella*, in RIF group vs. *Lactobacillus* abundances similar in RIF and control groups
Chen 2021, [38]	China	94 infertile women undergoing IVF: 25 with CE 69 without CE	EF (IUI catheter)	Women affected by chronic endometritis (CE): Lower clinical pregnancy rate compared to non-CE subjects High abundances of *Actinobacteria* and *Fusobacteria* Higher relative abundances of *Gardnerella* in non-pregnant subgroup Similar relative abundances of *Lactobacillus* between pregnant and non-pregnant subgroups
Moreno, 2022 [29]	Spain, USA, Turkey, Canada, Japan, Mexico, Malaysia, Argentina	342 infertile women undergoing IVF	ET and EF (Cannula of Cornier and catheter, respectively)	*Lactobacillus* strongly correlated with LBR *Gardnerella, Klebsiella*, and *Streptococcus* significantly more abundant in non-pregnant patients *Klebsiella* and *Staphylococcus* correlated with clinical miscarriage
Reschini, 2022 [31]	Italy	53 women undergoing IVF	EF (Double-lumen embryo transfer catheter)	*Lactobacillus* most represented in 16 patients (30%) 90% *Lactobacillus* abundance in 4 patients (8%) Higher endometrial biodiversity among pregnant women
Bednarska-Czerwińska, 2022 [39]	Poland	142 infertile women undergoing IVF	EF (Endometrial swab)	22 bacterial strains identified: 11 physiological strains (57%), with *Lactobacillus* most common 8 pathological strains (33%), with *Enterobacteriaceae* most common 3 pathological and physiological strains
Sezer, 2022 [40]	Turkey	26 women with unexplained infertility vs. 26 controls (age-matched)	EF (Endometrial swabs)	QLac/TBM (cutoff of 70.5%) had sensitivity of 84.6% in the infertility diagnosis
Kitaya, 2022 [41]	Japan	117 women with history of RIF vs. 55 infertile women (non-RIF group)	EF (Endometrial curette biopsy)	NLDM: 61/117 (52.1%) RIF group 30/55 (54.5%) non-RIF group After lactoferrin supplementation: Higher clinical pregnancy rate and LBR Similar miscarriage rate between the improved * VS/EF microbiota group and the unimproved VS/EF microbiota group
Keburiya, 2022 [42]	Russia	130 infertile women undergoing IVF: 39 with the first attempt (group I) 27 with RIF following embryo transfer with ovarian stimulation (group II) 64 with RIF following frozen-thawed embryo transfer (group III)	ET (Embryo transfer catheter tips)	Group I: *Lactobacilli* in moderate or high concentrations and opportunistic microorganisms (*G. vaginalis*) in moderate or high titers did not significantly affect embryo implantation Group II: Lower frequency of *Lactobacilli*, and low and moderate concentrations of opportunistic microorganisms (obligate anaerobes) compared to the first group Group III: Moderate concentrations of *streptococci, enterobacteria*, and especially strict anaerobes and *Gardnerella*

Acronyms: IVF = in vitro fertilization; ET = endometrial tissue; EF = endometrial fluid; LBR = live birth rate; LDM = *Lactobacillus*-dominated microbiota (>90% *Lactobacillus spp*); NLDM = non-*Lactobacillus*-dominated microbiota (<90% Lactobacillus spp. with >10% of other bacteria) [24]; IUI = intrauterine insemination; RIF = repeated implantation failure; COS = controlled ovarian stimulation; LD = *Lactobacillus* dominant (according to this study [34], >90% of the microbiota represented by *Lactobacillus* and *Bifidobacterium*); ART = assisted reproductive technology; CE = chronic endometritis; QLac = quantitative of *Lactobacillus*; QLac/TBM = ratio between QLac and total bacterial mass; vs. = vaginal secretions. * The increase (10% or more) in Lactobacillus species in microbiota composition [41].

### 2.3. Microbiome Sampling Direct from the Uterine Cavity during Surgery

The *Lactobacillus* dominance has also been questioned in different studies in which endometrial sampling was achieved using surgical procedures with direct access to the uterine cavity (open or laparoscopic hysterectomy, hysteroscopy, cesarean section, etc.). 

Indeed, *Acinetobacter, Pseudomonas, Sphingobium,* and *Vagococcus* were most abundant in 80 endometrial samples from Chinese women undergoing surgery for conditions not related to infection [10]. Another study including 137 Chinese women detected *Moraxellaceae, Propionibacteriaceae, Pseudomonadaceae,* and *Streptococcaceae* in the uterine cavity [43]. Later, in 25 Italian females selected for hysterectomy for fibroids, *Acinetobacter, Cloacibacterium, Comamonadaceae,* and *Pseudomonas* were dominant in the endometrial microbiota [44]. Regarding sampling during cesarean section, Leoni et al. found a common endometrial microbiota composition among 19 European women [45]. The authors assumed that microbes such as *Acinetobacter, Corynebacterium, Cutibacterium, Escherichia, Staphylococcus,* and *Streptococcus* could establish an endometrial microbiome core, while *Lactobacillus* was found with an abundance rate below 16% [45]. In another study, endometrial microbiome analysis in 10 patients at the time of caesarean delivery showed *Escherichia, Acinetobacter, Lactobacillus*, and *Bacillus* as the most frequently detected taxa [46]. 

### 2.4. First Meta-Transcriptomic Analysis

**Figure 1:**
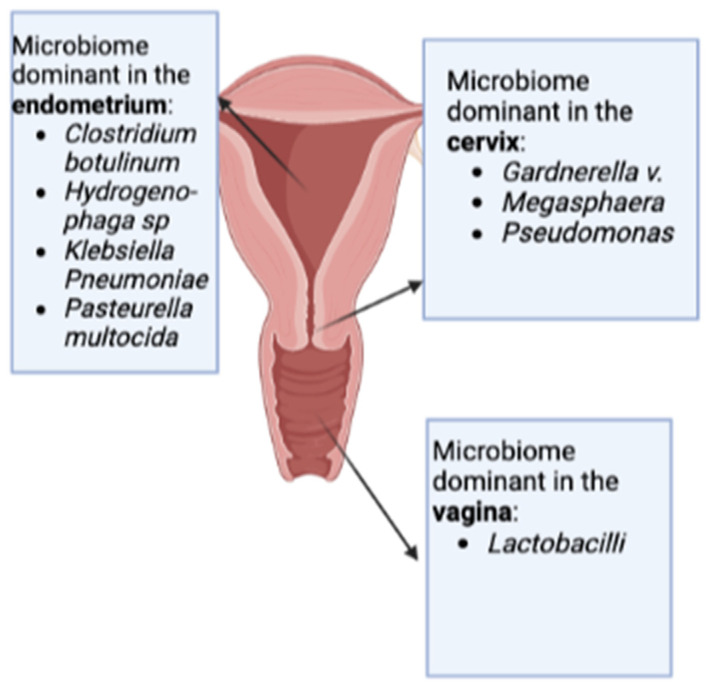
Composition of the endometrial microbiome according to a recent meta-transcriptomic study by Sola-Leyva et al. [47] and description of the cervical and vaginal microbiome by Chen et al. [10].

More recently, Sola-Leyva et al. [47] analyzed the uterine microbiome in seven healthy women using the meta-transcriptomic technique. The hypothesis was that DNA sequencing could detect live and dead microbes whose genetic material persisted, while RNA detection (transcriptome) could find only microorganisms still alive in the endometrium. They found 85% of bacteria, 10% of fungi, 5% of viruses, and 0.3% of archaea; among the bacteria, they found that, at least at the level of live microorganisms, *Lactobacillus* were not predominant in the endometrium of healthy young women in the mid-luteal phase, while *Clostridium botulinum, Hydrogenophaga* sp., *Klebsiella pneumoniae,* and *Pasteurella multocida* were the most abundant microorganisms, as shown in Figure 1.

### 2.5. Age and Hormonal Influence on Endometrial Microbiome Composition

The contrasting findings in terms of endometrial microbiome compositions can be explained not only by the different sites and methods of sampling, but also by the physiological changes in bacterial communities over the female lifespan. Indeed, several factors, such as age, hormonal changes, ethnicity, and intrauterine devices, can modulate the microbiome [48]. 

Regarding the age factor, Wang et al. studied endometrial and vaginal microbiomes in 145 women and found that in the uterine cavity the “alpha diversity” (e.g., microbial diversity within a single sample) was higher in the youngest women and tended to be slightly lower with advancing age. Conversely, the “beta diversity” (e.g., variability between communities) was lower among patients younger than 20 years old, while the greatest interindividual differences were found in patients aged 41–60 years [49]. 

**Figure 2:**
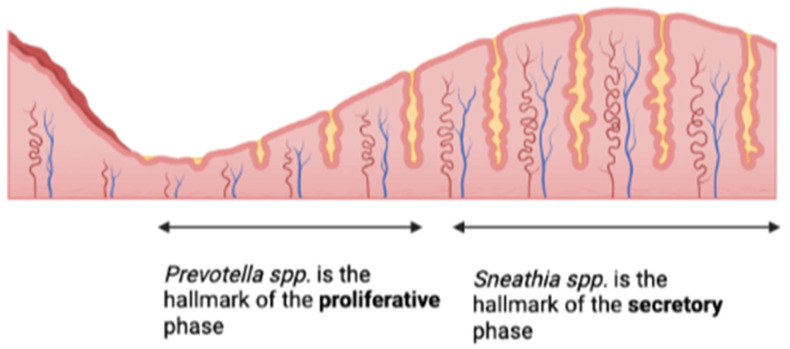
Endometrial microbiota changes under hormonal influence in different phases of the menstrual cycle [50].

Nevertheless, the endometrial microbiome can be influenced by hormonal changes during different menstrual cycle phases. Indeed, according to Pelzer and colleagues, *Prevotella* spp. could be considered the hallmark of the proliferative phase, while *Sneathia* spp. could be considered the hallmark of the secretory phase, as shown in Figure 2 [50]. In another study, *Lactobacillus* seemed to be detected in lower proportions after menstruation and to progressively increase during the follicular phase, with a peak during the luteal phase [34]. In addition, the endometrial microbiome seemed not to be influenced by hormonal changes during the phase of endometrial receptivity, as no differences were found among bacterial communities in endometrial samples collected 2 and 7 days after the luteinizing hormone (LH) peak [24]. Finally, according to the latest publications, the endometrial microbiome is more transcriptionally active in the mid-secretory phase compared to the proliferative one [47]. 

In addition, endometrial microbial composition can also change during treatment with exogenous hormones. Oral contraceptives and intrauterine devices, such as copper and levonorgestrel-releasing systems, seemed to change vaginal microbiome composition [51,52]. Similarly, it has been demonstrated that exogenous progestin induced decreasing diversity in *Lactobacillus* spp. phylotypes [50], while controlled ovarian stimulation and exogenous progesterone could modify both the diversity of bacterial taxa and bacterial abundance in the vaginal and endometrial microbiota [28].

## 3. Endometrial Receptivity and Immune Tolerance in Pregnancy

### 3.1. Introduction

There are several factors that contribute to a successful pregnancy, and embryo implantation is probably one of the most complicated processes, due to the intricate regulation of combined mediators, such as cytokines, lipids, adhesion molecules, and growth factors. To provide implantation, the endometrial tissue undergoes morphological changes during the mid-secretory phase of the menstrual cycle, also known as the implantation window. Then, the endometrium becomes “receptive” to guarantee the blastocyst’s attachment to the endometrial epithelial cells and to lead the invasion of the endometrial stroma and vasculature [53]. After implantation, because of the bidirectional cellular transport across the maternal–fetal interface, fetal antigens and fetal MHC molecules, which are a combination of both mother (self) and father (foreign), are constantly exposed to maternal circulation. Nevertheless, no maternal immune response against the fetus is triggered. 

### 3.2. Immune Cells and Cytokine Involvement According to Reproductive Phase and Gestational Age

The mechanisms of this immune tolerance during pregnancy have been largely investigated. Surprisingly, the immune system is not totally quiescent but in a constant modulation during the entire pregnancy, involving cellular populations that differ according to the different gestational periods, as summarized in Table 2. 

### 3.3. Uterine NK Cells

Other important immune agents include uterine NK (uNK) cells, which account for 60~90% of the decidual immune cells in the early gestational age and then decrease in the middle and late gestation periods [71,72]. The role of peripheral NK cells is the cytolysis of cells lacking self MHC-I, so in the uterus these cells could potentially be responsible for fetal rejection [73]. However, thanks to the different cell-surface receptors expressed by uNKcells and atypical MHC expression by the fetal trophoblast, the cytolysis is inhibited [74,75]. Nevertheless, during implantation, uNK cells are involved in the process of uterine spiral artery remodeling, which is also assisted by endometrial chemokines, such as LIF and IL11, and by cytokines produced by CD8^+^ T-cells, such as IL-8 and interferon γ (IFNγ) [55,63]. 

The activity of macrophages and uDCs, as described in Table 2, consists of suppressing local inflammatory responses by CD8^+^ T-cells against fetal antigens [76], inducing apoptosis of the CD8^+^ T-cells thanks to Trp depletion and promoting CD4^+^ T-cell differentiation into Treg cells [77]. 

### 3.4. Fetal Escape from Alloantigen Response

There are different mechanisms by which the embryo can escape from the alloantigen immune response. As previously explained, most of the polymorphic MHC class Ia antigens are lacking on the surface of trophoblast cells. Similarly, MHC class II antigens are not expressed. In this context, there is a general down-regulation of most of the MHC class Ia and class II molecules just before implantation occurs [78]. However, the maternal immune tolerance is also maintained when the paternal MHC is artificially re-expressed [79]. This could be explained by the prevalent indirect allogen recognition mediated by maternal antigen-presenting-cells (APCs), bypassing the direct recognition of intact fetal MHC molecules [80,81]. In the uterine environment, thanks to the decidualization process, the maternal APCs, and especially DCs, reduce in number and the remaining ones are less specialized during T-cell activation [60,82,83]. Another factor directly secreted by the embryo is preimplantation factor (PIF); it was demonstrated in several studies that it can modulate immune responses at different levels [84]. It has a major effect on the adaptive immune responses, by binding ligands to CD14 monocytes and neutrophils and to T- and B-cells, enhancing the Th2/Th1 cytokine ratio, and also by reducing IFNγ and stimulating IL-10 secretion. PIF also appears to modulate gene expression, thereby modifying oxidative stress, protein misfolding, and platelet activation [84]. Furthermore, PIF has an effect on the innate immune response, activating mainly the Toll-like receptor (TLR)-4 mediated NALP3 inflammasome complex, reducing IL-1β, IL-18, and IL-33 [85,86] and blocking the release of nitric oxide by macrophages activated by lipopolysaccharides (LPS) [87].

## 4. How Could Microbiota Modulate Immune Tolerance during Pregnancy?

### 4.1. Endometrial Microbiome during Embryo Implantation

In recent years, different reviews have summarized the current literature, with the aim of highlighting the endometrial microbial alterations in infertile women [29,48] and in women affected by gynecological disorders, such as endometriosis, endometrial hyperplasia, endometrial cancer, and chronic endometritis [88]. Compared to these previous studies, one of the objectives of our review was to emphasize the role of the endometrial microbiome not only in reproductive and obstetrical disorders, but mainly in physiological processes, such as the fine regulation of the immune balance needed for implantation.

Therefore, thanks to knowledge of the gut and in vitro and in vivo models, a hypothesis could be postulated. First, uterine bacteria or bacterial fragments can influence endometrial receptivity, inducing an inflammatory immune response. Indeed, endometrial epithelial cells represent a physical barrier blocking pathogen invasion, and they can produce antimicrobial peptides (AMPs), which have been linked to key regulatory processes in implantation [89]. In addition, it was demonstrated in a study on human endocervical epithelial cell cultures that commensal bacteria can induce membrane-associated mucin gene expression (MUC1, MUC4, and MUC16) and stabilize the adherent junctions and tight junctions [90]. 

### 4.2. Role of Bacteroides Fragilis

Relevant evidence was found regarding the role of *Bacteroides (B.) fragilis*. *B. fragilis* is an important Gram-negative anaerobic bacterium commonly found in the lower gastrointestinal tract; however, as stated previously, it belongs to the endometrial microbiome in non-pregnant women [91]. In germ-free mice colonized with *B. fragilis* strain NCTC9343, this bacterium has been shown to increase the CD4^+^ population thanks to polysaccharide A (PSA) secretion. Furthermore, PSA activated a signaling link to TLR2 that led to the differentiation of Th1 cells and the establishment of an appropriate Th1/Th2 balance [92]. For this reason, it could be hypothesized that PSA expressed by *Bacteroides* plays a role in endometrial receptivity due to the immunomodulatory effect. Recently, another study demonstrated in a mouse model that PSA can enhance TLR2 activation at the intestinal mucosal barrier and promote Foxp3+ Treg cells for immunologic tolerance [93]. 

### 4.3. Role of Lactobacilli

Another notable component of the endometrial microbiome in healthy women of childbearing age, although not representing more than 90% of the described bacterial taxa, as in the lower genital tract, is *Lactobacillus*. It is well known that lactic acid can inhibit the production of pro-inflammatory cytokines and chemokines through Toll-like receptor (TLR) activation [94,95] Furthermore, *L. delbrueckii* and *L. rhamnosus* seem to decrease the expression of HLA-DR, CD86, CD80, CD83, and IL-12 and to increase the expression of IL-10, IL-2, and IDO in immature DC cultures derived from healthy patients [96]. In conclusion, *Lactobacilli* could have a tolerogenic effect on the phenotypes of DCs during the maturation process. Similarly, another group demonstrated that *L. rhamnosus* GR-1 could inhibit pro-inflammatory gene expression in bovine endometrial epithelial cell cultures pre-treated with *Escherichia coli*, while an insignificant difference was found in the group treated with *L. rhamnosus* GR-1 alone [97]. Therefore, lactic acid could be an important immune modulator, acting at different levels in the immune response [98]. In particular, several studies on the potential role of *Lactobacilli* in preventing preterm birth in animal models have been conducted. Yang et al. found that the intraperitoneal administration of the *Lactobacillus rhamnosus* GR-1 supernatant in pregnant CD1 mice previously treated with an intrauterine injection of LPS via minilaparotomy significantly reduced the rate of preterm-birth (PTB) induced by LPS [99]. These findings were consistent, reporting decreases in anti-inflammatory cytokines generally related to human and murine PTB, in maternal plasma, myometrium, placenta, and amniotic fluid samples [99]. Later, Kim et al. demonstrated that the suppression of proinflammatory cytokines by *L. rhamnosus* is due to its unique capability to produce heat-resistant but trypsin-sensitive factors in the human myometrial cell line exposed to LPS [100]. Another study evaluated the protective role of *Lactobacillus kefiri* (Lk48) on PTB. Indeed, the oral prophylactic administration of Lk48 before and during pregnancy effectively reduced LPS-induced PTB and stillbirth in a mouse model. Furthermore, 18 h after the LPS injection, no sign of inflammatory response was found in a histological analysis of endometrial tissues of Lk48-treated mice as compared to controls. Moreover, significantly lower percentages of CD8^+^ T-cells were found in a flow cytometry analysis of uterus and decidua samples from Lk48-treated mice [101]. 

## 5. Endometrial Microbiota Dysbiosis and Adverse Reproductive Outcomes

In recent years, there has been a growing interest in the impact of the endometrial microbiota on reproductive outcomes.

### 5.1. Recurrent Implantation Failure (RIF)

By analyzing a great number of studies on the topic, which are reported in detail in Table 1, we concluded that a unique consensus on the endometrial microbes related to RIF has not been established yet and that a further evaluation to correlate the inflammatory uterine environment, which is known to be involved in female infertility [102,103] and endometrial dysbiosis, is needed (see Section 2.2).

### 5.2. Recurrent Pregnancy Loss (RPL) 

Recurrent pregnancy loss (RPL), defined as the loss of two or more pregnancies [104], is a pregnancy complication with an incidence range of 2–5% in couples trying to conceive [105].

Although several risk factors have been associated with RPL, in more than 50% of women RPL is idiopathic [106]. 

Peuranpää et al. studied endometrial and vaginal microbiota in 47 women with a history of two or more consecutive miscarriages, of whom 36 had experienced unexplained RPL. They found that in RPL patients *Lactobacillus crispatus* was significantly less abundant and that *L. jensenii* and *G. vaginalis* were more abundant compared with the control group, while no significant difference was found for *Escherichia coli, Blautia* spp., or *Faecalibacterium* spp. [107]. Furthermore, *Lactobacillus iners* represented the dominant endometrial bacterium in RPL patients, and, from the fungal analysis, *Candida parapsilosis* was detected only in endometrial samples of RPL patients, with an average relative abundance rate of 18.2% [107]. Then, another study evaluated 25 RPL patients and found significant differences in the beta diversity levels of microbiota from uterine lavage fluid samples between cases and controls [108]. In addition, the microbiota of the endometrial tissue of the RPL group showed a lower abundance of *Lactobacillus spp*., and, among the *Proteobacteria, Acinetobacter* spp. were predominant [108]. Interestingly, when analyzing human Th1/Th2/Th17 cytokines in uterine lavage fluid, the expression levels of IFN-γ and IL-6 were significantly inferior in the RPL group and statistically significant correlations between *Aliihoeflea* and IL-17A, *Acinetobacter* and IFN-γ, *Serratia* and TNF, and *Staphylococcus* and *Serratia* and IL-6 were found [108]. Lastly, in a study by Masucci et al., endometrial and vaginal microbiota were analyzed in 40 RPL patients, of whom 15 were HLA-DQ2/DQ8-positive and 25 were HLA DQ2/DQ8-negative, to investigate the pathogenic effect of celiac disease on microbiota [109]. A higher endometrial microbial diversity was found among control groups compared to RPL, with or without genetic predisposition. Furthermore, the *Lactobacillus* abundance levels were higher in RPL patients (66.2% in HLA-DQ2/DQ8-negative and 72.62% in HLA-DQ2/DQ8-positive patients) compared to controls (48.4%), and *L. iners* was much higher in both subgroups of RPL women compared to the control group, while *L. acidophilus* was almost absent [109]. Finally, *Gardnerella* was more represented in RPL HLA-DQ2/DQ8-positive patients, and *Atopobium* spp. was detected only in RPL patients, with or without genetic celiac predisposition [109].

### 5.3. Preterm Birth (PTB)

Analyzing the most recent literature on the correlation between microbiome composition and PTB, great attention has been paid to vaginal microbiome alterations rather than to the endometrial environment. According to a recent overview, conflicting results have been reported due to methodological heterogeneity, the ethnic backgrounds of the included patients, and the associated risk of PTB [110]. To our knowledge, there are no available studies that have directly related endometrial microbiome alterations to PTB. Indeed, due to the cervical shortening in preterm labor or the preterm premature rupture of membranes, the endometrial and vaginal environments have often been considered comparable. 

## 6. Methodological and Sampling Considerations

### 6.1. Introduction

The last aim of this review is to provide some practical guidelines for physicians working on endometrial microbial analyses. Indeed, the limitations of the previously mentioned studies have already been noted in a recent review [88]. The most ambitious—but fundamental—objective is to standardize the methods used to compare the results of future studies. There are two major challenges in obtaining such results: first, the low microbial biomass of the uterus (especially when compared to the cervix and vagina); second, the high risk of contamination during sampling. To overcome these intrinsic limitations of the uterine microbiome, choosing the correct sampling method is key to reducing this risk. As an alternative to the conventional *Pipelle*, the preferred instruments used to minimize contamination are the following:

- A double-sheathed embryo transfer catheter;
- An intrauterine insemination catheter;
- A transcervical sheathed brush device.

Lastly, to reduce contamination, the use of appropriate controls is fundamental [111,112]. As has been well explained by Eisenhofer et al. [112], three types of negative controls are recommended to adequately detect contaminants:

(1) Sampling blank controls, to allow the detection of contaminant DNA introduced during the sampling procedure (e.g., swabs and plastic bags) and any tools used to store or to transport the samples from the sampling site to the laboratory;
(2) DNA-extraction blank controls, to monitor contaminant DNA contents in the process from extraction to sequencing;
(3) No-template amplification controls, to monitor contaminant DNA in the process of library preparation and sequencing.

On the other hand, two types of positive controls are recommended:

(1) DNA-extraction positive controls, to monitor DNA-extraction efficiency: the advice is to use a positive control of known concentration that is relevant to your study and experimental questions;
(2) Positive amplification controls, using a titration of DNA from a known organism type to be processed during the library preparation step.

### 6.2. Fluid or Tissue?

In 2018, Liu et al. [113] raised doubts regarding whether the endometrial fluid microbiome and endometrial tissue microbiota fully correspond. Specifically, they identified fewer taxa per 1000 sequencing reads from fluid compared to tissue samples. Further, they reported lower diversity in fluid compared to tissue samples. In conclusion, the endometrial fluid only partially reflects the endometrial microbiome, and a possible explanation for this finding is that the bacteria on the superficial layer of the endometrium are different from those deeper in the endometrial tissue, including the stroma. Thus, it is suggested to include both when performing a study on the endometrial microbiome.

### 6.3. Storing Methods

Snap-freezing and direct freezing at −80 °C are the gold standards [111]. As an alternative, it is suggested to use storage media that can stabilize nucleic acids at higher temperatures. It is suggested to avoid repeated sequences of “freezing–thawing”.

### 6.4. Study Planning

Moving from sampling methods to study planning, Molina et al. [111], in 2021, described a list of considerations to follow before starting a study on endometrial microbiota.

Regarding the study design, these are four strong suggestions:

1. To take samples at identical time points;
2. To take samples longitudinally from the same subject (especially if in pregnancy);
3. To use PERMANOVA for the statistical analysis;
4. To make publicly available the following details: the characteristics of the study population, the sample type, the collection method, and the data processing and analysis workflow.

Regarding the study population, especially in those studies involving reproductive outcomes, it seems particularly important to distinguish the phase of the menstrual cycle, since the uterine microbiota undergoes deep changes due to hormonal input [47]. In this context, it seems particularly relevant to take samples at identical time points, as specified above. Further, it also seems relevant to specify parity, since it has been proved that nulliparas and multiparas present different uterine microbiota compositions [48].

### 6.5. Ethical Considerations

Pain and discomfort frequently accompany the insertion of a catheter in the uterine cavity, both during and after the procedure. Taking nonsteroidal anti-inflammatory drugs, such as ibuprofen, before the procedure can significantly reduce the discomfort. Endometrial sampling should not be performed in case of pregnancy. All patients with the potential for pregnancy should be tested prior to proceeding with endometrial sampling.

## 7. Conclusions

In the last two decades, an increasing amount of evidence regarding uterine microbiota composition has become available, but a consensus on the endometrial core composition and its relation to the physiological mechanisms that lead to a successful pregnancy has still to be reached. Furthermore, more studies with the aim of unraveling the link between the uterine microbiota, maternal immune response, and clinical reproductive and obstetrical outcomes are needed. The future challenge will be to analyze microbiota in larger patient cohorts and according to standardized methods for sampling, comparative analysis, and interpretation. The aim is to reduce the large amount of variability in results and to understand the microbial roles in various clinical disorders.

## Figures and Tables

**Table 2:** Immune cells and cytokine involvement according to the reproductive phase.

Timing	Immune Cell Assets	Cytokines
Mid-ovulatory		Increasing levels of: IL-6 IL-8 IL-15 GM-CSF TNF-α [54]
Implantation	Increasing numbers of NK cells [55] (progesterone-mediated). Increasing numbers of Treg cells [56,57]. Shift to M2 macrophages [58], induced by growth factors released by the trophoblast uDCs expressing fewer CD83 and CD86 costimulatory molecules [59]	Thanks to progesterone, decreasing levels of GM-CSF and IL-1 [60] and increasing levels of IL-8 [60]. Thanks to estrogen, increasing levels of: CCL-3 CCL-4 CCL-5 [61] Thanks to M2 macrophages, increase in PGE2 and IL10 [62]. For uterine spiral artery remodeling, increase in: LIF IL11 IL-8 IFNγ [55,63]
First and second trimester	Treg effects [64,65,66,67,68,69] (peak during second trimester): Decreasing: Th1/Th17 responses B-cell proliferation Antibody production NK cytotoxicity Increasing: Decidual T-cells and Macrophage levels	Treg effects [64,65,66,67,68,69] Increasing levels of: IL-10 LIF TGF-β HO-1 IL-25 [70] IL-4 IL-10

Acronyms: IL = interleukin; GM-CSF = granulocyte–macrophage colony-stimulating factor; TNF = tumor necrosis factor; NK = natural killer; Treg = regulatory T; M2 macrophages = wound-healing phenotype of macrophages; uDCs = uterine dendritic cells; PGE2 = prostaglandin E2; LIF = leukemia inhibitory factor; IFNγ = interferon γ; CCL = chemokine C-C motif ligand; TGF = transforming growth factor; HO = heme oxygenase.

## Data Availability

No new data were created.
